# Time to initiation of dialysis and length of stay in hospitalized patients with kidney damage: a cross-sectional study

**DOI:** 10.1590/1516-3180.2023.0365.R1.03072024

**Published:** 2024-12-20

**Authors:** Douglas Vieira Gemente, Marcelo Rodrigues Bacci

**Affiliations:** I Nephrology Division, Hospital Santa Marcelina, São Paulo (SP), Brazil; Centro Universitário Faculdade de Medicina do ABC (FMABC), Santo André (SP), Brazil.; II Assistant Professor, General Practice Department, Post-Graduation Program, Centro Universitário Faculdade de Medicina do ABC (FMABC), Santo André (SP), Brazil.

**Keywords:** Delivery of Health care, Dialysis, Renal insufficiency, chronic, Kidney care, Public health expenses, Length of hospitalization, Brazilian unified health system

## Abstract

**BACKGROUND:**

Universal healthcare is a cornerstone of Brazil’s public health system. However, delayed diagnosis and treatment of chronic kidney disease (CKD) remain substantial issues. The scarcity of outpatient dialysis facilities contributes to extended hospital stays. This study aimed to examine how the time to dialysis initiation (TID) impacts mortality in patients with renal disease.

**OBJECTIVES:**

This study aimed to evaluate the correlation between variables affecting TID and mortality in hospitalized patients with renal disease.

**DESIGN AND SETTING:**

A cross-sectional study was conducted at Santa Marcelina Hospital in São Paulo.

**METHODS:**

This cross-sectional study was conducted in a tertiary hospital, involving adults with kidney disease who were referred to the emergency department between 2014 and 2017. Primary outcomes included TID and mortality rates.

**RESULTS:**

Among the 402 patients studied, the average age was 58.6 years, and 59.4% were men. The median hospital stay was 44.5 d. Notably, 28.1% of the patients began dialysis under emergency conditions. Diabetes and hypertension were the most prevalent causes of renal disease. A positive correlation was found between age and TID (P = 0.007).

**CONCLUSIONS:**

Primary care in Brazil often fails to effectively detect and manage CKD, leading to a higher incidence of emergency dialysis, particularly among older adults. This delay correlates with increased mortality rates. Older age is associated with delayed TID, prolonged hospital stays, and consequently higher mortality. These findings highlight the need for better primary care to effectively manage CKD and reduce hospitalization and mortality.

## INTRODUCTION

Universal access to healthcare is a significant topic of debate worldwide. Both politicians and the general population recognize the importance of creating a sustainable and cost-effective system to support the treatment and prevention of serious health conditions. Brazil, a country of continental proportions, established its Unified Health System (SUS) in the 1980s.^1^ The system ensures that every Brazilian citizen has the right to receive medical treatment, not only for chronic conditions but also for preventive care, medication distribution, and diagnostic testing. Since its implementation, the SUS has improved access to healthcare for low-income individuals, particularly in areas such as prenatal care and vaccination. However, challenges remain, including inequitable coverage, conflicting ideologies and goals, and limitations in financing, infrastructure, and human resources.^2^

Chronic kidney disease (CKD) is a public health issue with increasing prevalence over recent decades. Its leading causes, hypertension and diabetes, continue to grow in prevalence. To understand the economic burden of this disease, the 2022 annual report from the United States Renal Data System indicated that in 2020, total fee-for-service expenses for all beneficiaries reached $85.4 billion, accounting for 23.5% of all Medicare expenditures.^3^ Brazil has the third-largest population of patients with end-stage renal disease on hemodialysis globally, with the SUS primarily supporting this care. Despite a budget allocation of $ 80 million for initial dialysis care, the system struggles to prevent the progression of CKD.^4^ Despite the growing number of patients initiating dialysis, estimated at 133,464 by the Brazilian Nephrology Society, these figures do not capture the full burden of in-hospital dialysis costs incurred by public hospitals under the SUS and by the private healthcare sector.^2,5^ The need for hospitalization to initiate renal replacement therapy increases the risk of death and raises the overall cost of CKD care. However, data on these factors not well-documented in Brazil.

## OBJECTIVE

This study aimed to evaluate the correlation between variables affecting the time to initiate dialysis (TID) and mortality in hospitalized patients with renal disease at a major hospital in São Paulo.

## METHODS

This cross-sectional study was conducted at a major tertiary hospital in São Paulo, Brazil, between 2014 and 2017. All medical records of hospitalized adult patients admitted during this period with kidney damage and no previous renal replacement therapy were analyzed. A review of these records revealed the study participants’ demographics and baseline clinical characteristics. No personal information was shared during the review of medical records. A flowchart explaining the patient selection process is provided in the Supplemental Material.

The primary diagnosis at admission was considered to address the etiology of renal dysfunction, preexisting conditions, and patient demographic information. In Brazil, patients with a permanent indication for renal replacement therapy are discharged from the hospital only when an external dialysis facility has seats available to continue treatment. The TID in this study referred to the time when the patient was admitted to the external dialysis facility and not to the in-hospital indication of renal replacement therapy. In the public system, external dialysis facilities must have available seats for patients. This study followed and revised the checklist principles for items that should be included in reports of observational studies (STROBE).

Kidney damage evaluation followed the Kidney Disease: Improving Global Outcomes (KDIGO) guidelines.^4^ Acute kidney injury (AKI) diagnosis followed the 2012 KDIGO criteria, where a significant rise in serum or plasma creatinine of at least 0.3 mg/dL within 48 h, or suspected to have occurred within the preceding 48 h, compared to baseline serum creatinine levels, was used to make the diagnosis.^6^ For individuals who developed hospital-acquired AKI, the baseline reference was a consistent serum creatinine level obtained during the patient´s hospital stay before the diagnosis of AKI. Evaluation of the medical records was triggered by a call to the nephrology team in the emergency room and involved tracking the patient’s clinical origin and kidney function. For individuals admitted from the community, the study team searched for reference serum creatinine levels in the following order of preference: the most recent value recorded within 3 months before hospital admission. If this was not accessible, the value obtained within 3 to 12 months prior to hospital admission was considered. If this was also unavailable, serum creatinine levels upon hospital admission were recorded. If the patient had no renal abnormalities, their medical record was excluded from the evaluation.

The evaluation of CKD was based on a sustained decrease in the estimated glomerular filtration rate for at least 12 weeks, according to KDIGO guidelines for CKD management.^4^ Persistent albuminuria or structural kidney alterations observed in imaging tests were also considered for CKD diagnosis and staging. The etiology of kidney failure was classified into six categories: glomerulonephritis, arterial hypertension, diabetes, oncology-associated, sepsis-related, and other causes, such as drug-induced renal dysfunction. The study team did not influence the indications for renal replacement therapy, which were solely determined by the hospital nephrology staff. Discharge or transfer to an external dialysis facility was also managed by the hospital team. The primary outcome was the TID in days after the diagnosis of renal dysfunction, as well as death from any cause. The length of stay (LOS) was analyzed as a secondary outcome, correlated with death.

Descriptive analyses were conducted by measuring central tendency and dispersion. Proportion measures were used to describe qualitative variables. After confirming the normality of the variables using simple histograms, parametric tests were performed. The TID was compared between the groups (survivors and deceased) using the Student’s t-test. LOS and TID were evaluated using linear regression and Pearson’s correlation tests. Multivariate analysis was performed with the best baseline characteristic variables against TID and LOS. An alpha error rate of 5% was assumed. The statistical software used was XLStat for Windows. The study was approved on 03/13/2019 by the Faculdade de Medicina do ABC (FMABC) review board, under reference number 94759218.4.0000.0082.

## RESULTS

The study population included 402 patients with complete medical records during data collection. Most patients were between 18 and 60 years of age, and 60% of men had kidney damage during the period analyzed. There was significant variability in the LOS, ranging from 3 to 410 d, with a median LOS of 44.5 d. The majority of the patients (78.6%) stayed for a maximum of 30 d. The prevalence of hypertension and diabetes as causes of renal dysfunction was higher than that of other categories, as shown in **Table 1**. Approximately 52.5% of the patients had previously consulted a nephrologist, and 53.3% had kidney damage identified in the emergency room.

**Table 1:** Demographics and clinical characteristics of the sample

	n	%
**Age (years)**		
18–59	208	52
60–79	174	43
> 80	19	5
**Sex**		
Men	234	60
Women	168	40
**Length of stay (days)**		
01–30	316	78.6
31–60	41	10.2
> 60	45	11.2
**Admission creatinine (mg/dL)**		
< 1.0	97	24.2
1.0–3.0	176	35.3
> 3.0	163	40.5
**Renal dysfunction etiology**		
Diabetes	96	24
Hypertension	165	41
Sepsis	20	5
Cancer	25	6
Glomerulonephritis	80	20
Other causes	16	4

**Figure 1:**
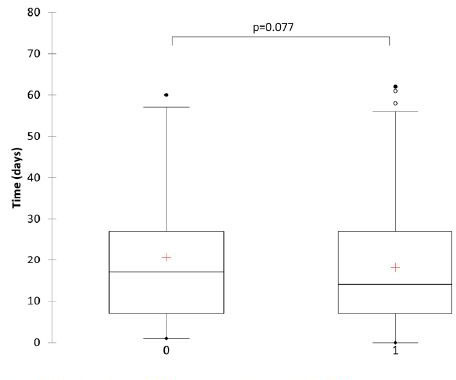
TID and death in patients hospitalized with kidney damage.

**Figure 2:**
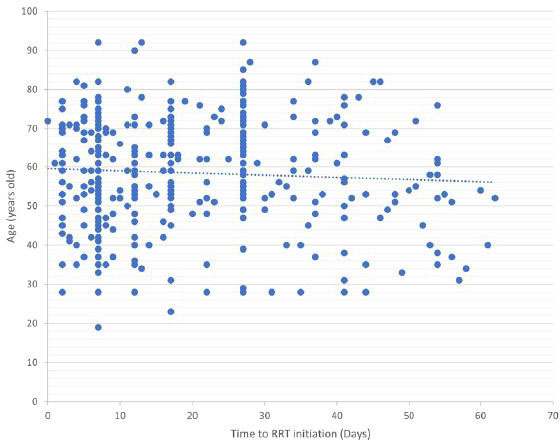
Univariate analysis between age and time to initiation of dialysis in hospitalized patients.

The comparison between TID and death using the Student’s t-test was not statistically significant, as shown in **Figure 1**. Pearson’s univariate analysis revealed a positive correlation between age and TID (P = 0.007) (**Figure 2**).

0 = Alive; 1 = Dead; TID = time to initiate dialysis.

RRT = renal replacement therapy. * Pearson´s correlation test.

Multivariate analysis was used to evaluate the possible explanatory variables tested against dialysis initiation time (**Table 2**). None of the patient characteristics (age, prior nephrologist consultation, kidney damage etiology, or the patient’s origin) were significantly correlated. Age was the most influential factor in the TID model.

**Table 2:** Multivariate analysis between selected variables and time to initiate dialysis vs. length of stay

Variable	P
Age	0.226
Previous nephrologist consultation	0.442
Origin of the patient (ER x Community)	0.365
Kidney damage etiology	0.801

ER = emergency room.

**Figure 3:**
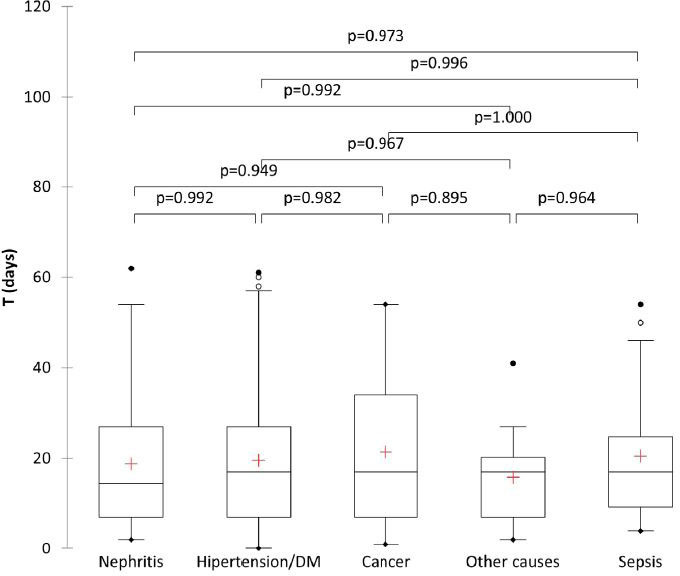
Etiology of renal dysfunction and time to initiation of dialysis.

TID and LOS were tested against the etiology of kidney damage in each of the five categories (diabetes and arterial hypertension, oncology-associated, glomerulonephritis, sepsis-related, and other causes), as shown in **Figure 3**. All causes analyzed using the Kruskal–Wallis test showed a P value of 0.822. Individual etiologies related to TID and LOS were also analyzed using the Steel–Dwass–Critchlow–Fligner test (**Figure 3**).

LOS = length of stay; TID = time to initiate dialysis; DM = diabetes; * Individual analysis performed using the Steel–Dwass–Critchlow–Fligner test; **Full model analysis performed using the Kruskal–Wallis test (P = 0.822).

## DISCUSSION

In this cross-sectional study conducted at a tertiary hospital in São Paulo, patients with renal dysfunction admitted for treatment were evaluated to identify the variables that contributed to their prognosis and their influence on TID and LOS. During the observation period, most admitted patients had previously consulted a nephrologist, and age was identified as the primary factor contributing to delayed TID in this cohort.

Over the past 50 years, CKD has transitioned from a neglected disease to a critical public health issue.^7^ However, in developing countries, there is a lack of oversight regarding the management of renal disease, from screening to dialysis or organ transplantation. In Brazil, studies have reported a gap in primary care physicians’ knowledge of kidney disease identification and diagnostic screening.^7, 8, 9^ This situation is exacerbated in the Brazilian public health system (SUS) due to the uneven distribution of nephrologists across the country, with more developed regions and cities having a higher concentration of specialists, whereas less wealthy areas face delays in accessing nephrology care. The hospital where the study was conducted is located in São Paulo, Brazil’s largest city, but in a less affluent neighborhood, receiving patients from distant regions with limited access to medical specialists. In our sample, many patients had not previously consulted a nephrologist. Moreover, the average LOS for patients with kidney damage was 44 d, which is consistent with the findings of Cruz et al., who reported that late referral to a nephrologist was associated with higher mortality and prolonged hospital stays.^10^

A late indication for dialysis might influence the LOS. In this cohort, the hospital staff determined dialysis indications according to standard clinical and metabolic criteria. Neurological symptoms, such as uremia, refractory metabolic acidosis, hyperkalemia, and fluid overload, are the most critical clinical indications for urgent dialysis initiation. However, the indication for dialysis can be subjective, and other criteria, such as the patient’s age, diuresis, and the intuition of a reversible cause of kidney damage might delay this decision. A strict clinical approach to renal dysfunction rather than early initiation of dialysis may increase the risk of an urgent start. A recent review showed that the cost of urgent dialysis was USD 6,092 per patient over 6 months.^11^ In this cohort, 28.18% of the patients required urgent dialysis initiation during the observation period. As there was a significant proportion of patients without proper nephrology treatment before hospitalization, delayed TID negatively affected patients needing more time to stabilize and who lacked definitive vascular access, such as a venous fistula. From the data gathered, we identified a direct relation between longer LOS due to the late release of the outpatient hemodialysis bed and higher mortality, especially among older people. This finding was corroborated by Cruz et al., who reported that patients older than 70 years had a higher mortality rate when starting renal replacement therapy.^9^

This study has several strengths. It is the first Brazilian study to provide real-world evidence of delayed TID in an outpatient dialysis facility for hospitalized patients with end-stage renal disease. The leading causes of renal dysfunction in this cohort were consistent with the main etiologies of preventive care: arterial hypertension, diabetes, and sepsis, within the hospital environment. We did not have access to the individual costs for each patient to show the burden of delays in initiating external dialysis. This was a single-center study, and some findings might reflect the nephrology staff’s standard of care practices, which could have impacted dialysis indications. Critical care patients and those who underwent elective surgery were not analyzed, as this was not the main objective of this study. Data were retrieved from the electronic medical records. However, the study team manually inserted the data, which could have caused some inconsistencies, even with a double-check safety procedure for each data insertion.

## CONCLUSION

In summary, in this cross-sectional study, aging was positively correlated with late TID and longer LOS in patients hospitalized for renal disease. The number of external dialysis seats available for the public system must increase rapidly as the prevalence of CKD continues to rise in Brazil and worldwide. Despite being conducted in a single-center hospital, this study provides future directions for a broader, multicenter evaluation to understand all the variables involved in reducing LOS and contributing to early discharge for end-stage renal disease patients. Moreover, it highlights the importance of primary care in renal function screening, thereby reducing the burden on these patients during hospital care.
